# The Spread of Antibiotic Resistance Genes In Vivo Model

**DOI:** 10.1155/2022/3348695

**Published:** 2022-07-18

**Authors:** Shuan Tao, Huimin Chen, Na Li, Tong Wang, Wei Liang

**Affiliations:** ^1^School of Medical, Jiangsu University, Zhenjiang, Jiangsu Province, China; ^2^Lianyungang Clinical College of Jiangsu University, Lianyungang, Jiangsu Province, China; ^3^Bengbu Medical College, Bengbu, Anhui Province, China; ^4^Nanjing Brain Hospital Affiliated Nanjing Medical University, Nanjing, Jiangsu Province, China

## Abstract

Infections caused by antibiotic-resistant bacteria are a major public health threat. The emergence and spread of antibiotic resistance genes (ARGs) in the environment or clinical setting pose a serious threat to human and animal health worldwide. Horizontal gene transfer (HGT) of ARGs is one of the main reasons for the dissemination of antibiotic resistance in vitro and in vivo environments. There is a consensus on the role of mobile genetic elements (MGEs) in the spread of bacterial resistance. Most drug resistance genes are located on plasmids, and the spread of drug resistance genes among microorganisms through plasmid-mediated conjugation transfer is the most common and effective way for the spread of multidrug resistance. Experimental studies of the processes driving the spread of antibiotic resistance have focused on simple in vitro model systems, but the current in vitro protocols might not correctly reflect the HGT of antibiotic resistance genes in realistic conditions. This calls for better models of how resistance genes transfer and disseminate in vivo. The in vivo model can better mimic the situation that occurs in patients, helping study the situation in more detail. This is crucial to develop innovative strategies to curtail the spread of antibiotic resistance genes in the future. This review aims to give an overview of the mechanisms of the spread of antibiotic resistance genes and then demonstrate the spread of antibiotic resistance genes in the in vivo model. Finally, we discuss the challenges in controlling the spread of antibiotic resistance genes and their potential solutions.

## 1. Introduction

The spread of bacterial drug resistance and pathogenicity of bacteria impose substantial health and economic burden [1], and its wider implications present us with a growing healthcare crisis [2]. Antibiotic resistance genes (ARGs) can be vertically transferred and spread via horizontal gene transfer (HGT) through mobile genetic elements (MGEs) among bacteria [3]. Mechanisms mediating the horizontal transfer of ARGs include transformation, conjugation transfer, and transduction, membrane vesicles (MVs), and DNA packaged into virus-like particles [4]. Bacterial mobile genetic elements (MGEs), such as conjugative plasmids and integrative and conjugative elements (ICEs), have been highlighted as important vehicles for the dissemination of pathogenesis and antimicrobial resistance determinants [5]. Conjugative plasmids exhibit a wide host range and thus can shuttle ARGs between different genera, orders, and even phyla [6]. The sharing of genes through HGT contributes importantly to the global dissemination of antibiotic resistance genes (ARGs) [7]. HGT can occur in any environment, particularly when bacterial loads are high, for example, in soil, in wastewater treatment plants [8, 9], and in the gut microbiome of humans and animals based on the transfer-related genes carried on plasmids [10]. Most of the current knowledge of the spread of antibiotic resistance was obtained by in vitro or observational studies [11]. However, there remains limited knowledge in vitro to predict the horizontal transfer of antibiotic resistance genes, and the in vitro models may not correctly reflect the HGT of the resistance genes in vivo. Thus, further studies are needed to understand the horizontal transfer of antibiotic resistance genes in vivo [12].

In this review, we provided a brief overview of the dissemination modes and main transmission mechanisms of horizontal gene transfer of antibiotic resistance genes, introduced the spread of antibiotic resistance genes in vivo model, elaborated some current methods to control bacterial resistance, and described the future perspectives for antimicrobial resistance gene removal, thereby presenting a certain reference for the control of the spread of antibiotic resistance genes.

## 2. Dissemination Modes of Antibiotic Resistance Genes

The dissemination modes of drug resistance genes in pathogens can be performed by both vertical gene transfer (VGT) and horizontal gene transfer (HGT) [13]. VGT is transmitted in the generations. During the process of bacterial division, the drug resistance gene is transmitted from parent to offspring [14]. HGT breaks the boundaries of relatedness compared to VGT, enabling the exchange of genes between different species [15]. It has been shown that the HGT of bacterial drug resistance plays an important role in the evolution and spread of multidrug resistance [16]. HGT can be performed by transformation, transduction, and conjugation, of which conjugation is the most important mode, and this mechanism is widely found in bacteria [17]. Mobile genetic elements (MGEs) shared their genetic elements of resistance with other nonresistant bacterial species via HGT, which promoted the accumulation and dissemination of ARGs in Gram-negative and Gram-positive bacteria [18, 19]. When the drug resistance genes in bacteria accumulate to a certain extent, it is possible to form highly pathogenic super-bacteria resistant to most antimicrobial drugs, thus posing a serious threat to human health.

## 3. Mechanisms of the Horizontal Gene Transfer

**Figure 1:**
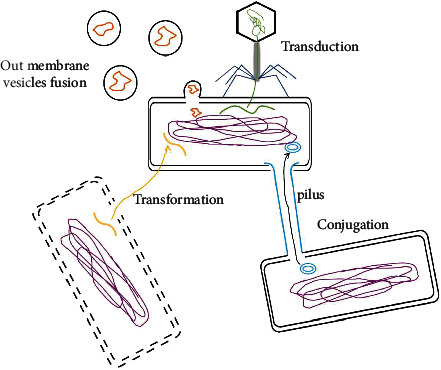
General ways of horizontal gene transfer. Conjugation, Transformation, Transduction, and Out membrane vesicles fusion. Yellow represents DNA fragments; blue represents conjugation elements (ICEs); orange represents membrane vesicles (MV).

Antibiotic resistance spreads among bacteria mainly through the horizontal transfer of antibiotic resistance genes (ARGs) [20]. Horizontal gene transfer (HGT) plays an important role in bacterial evolution and greatly facilitates the rapid spread of resistance genes [21]. The mechanisms of horizontal gene transfer mainly include conjugation, transformation, and transduction [22]. In addition, the role of membrane vesicles (MVs) has also been verified in HGT. See Figure 1.

### 3.1. Conjugation

Conjugation is the transfer of genetic material (such as plasmid DNA) from donor bacteria to recipient bacteria through direct physical cell-to-cell contact [23]. Conjugation is the most important way of horizontal transfer, and this mechanism is widely present in bacteria [24]. Conjugation is a contact-dependent process where mobile genetic elements, such as plasmids and integrating and conjugation elements (ICEs), are transported through a pilus or pore between bacteria close to each other [25]. Resistance genes can be transmitted through the conjugation between the same genus or different species. The spread of mobile genetic elements has been observed in commensal and opportunistic pathogens while colonizing the human gut [26]. Conjugation of plasmid-mediated antimicrobial resistance genes and the transmission of drug resistance pose a serious threat to human health [27]. The plasmids carrying carbapenemase resistance genes (such as blaKPC, blaNDM, and blaOXA-48) in Gram-negative bacteria can be rapidly transmitted to other susceptible bacteria by conjugation, which has become a major global health threat [28]. It has been reported that the plasmid encoding OXA-48 (carbapenem resistance) from *Enterobacter cloacae* may be conjugally transferred to other members of the Enterobacteriaceae family in the gastrointestinal tract [29]. Studies have demonstrated that the ICE-mediated drug resistance transmission mechanisms can also be found in Gram-positive bacteria, such as *Streptococcus* spp. [30].

### 3.2. Transformation

Transformation means that extracellular DNA from lysed donor bacteria is taken up by the recipient bacteria and integrated into their genomes so that the recipient bacteria can acquire new traits [31]. Extracellular DNA is mostly plasmid DNA and fragmented DNA released during active secretion or lysis by bacteria, often carrying ARG [32]. Acquired resistance through natural transformation is thought to occur frequently in many clinical bacterial species [33]. For example, *Neisseria gonorrhoeae*, *Vibrio cholerae*, and *Streptococcus pneumoniae* can acquire antibiotic resistance through transformation [34]. Studies have shown that *E. coli* can be transformed by plasmid DNA under natural conditions, indicating that *E. coli* can absorb DNA in the gut, and therefore, it can be considered that transformation can contribute to the transmission of ARGs [21, 35]. Fondi et al. [36] reported that the sequenced *Acinetobacter* plasmids lack the genes required for conjugative transfer, indicating that some drug-resistant plasmids of *Acinetobacter baumannii* are not disseminated by conjugation but possibly through the natural transformation pathway.

### 3.3. Transduction

Transduction uses mild bacteriophage as a carrier to transfer chromosomal and extrachromosomal DNA from the donor bacteria to the recipient bacteria so that the recipient bacteria can acquire new traits [37]. Phages can coexist with ARGs in the same ecological environment and the same bacteria, indirectly suggesting that phages may play a role in the spread of drug resistance genes [38, 39]. Resistance transduction is more common in *Staphylococcus aureus* [40]. Methicillin-resistant *Staphylococcus aureus* (MRSA) acquires resistance from other bacterial species conferring the mecA gene by phage-mediated transduction [41]. The Phage*φ*80*α* can not only mediate the transmission of penicillin and tetracycline resistance genes to the multidrug-resistant *S. aureus* strain USA300 but also mediate the transfer of resistance genes to the phage-unsusceptible *S. aureus* spp. [42, 43]. Transduction may occur in nature anytime, anywhere, and its role in the transmission of drug resistance is far beyond our imagination [44]. Experiments in mouse models have demonstrated that transduction is a driving force behind genetic diversity in gut-colonizing *E. coli* strains [45] and can promote the emergence of drug resistance in gut bacteria [46].

### 3.4. Other Mechanisms of the Horizontal Gene Transfer

Horizontal gene transfer can also be carried out through lysogenic conversion, transposition, and protoplast fusion [17]. Recently, the roles of membrane vesicles (MVs) in HGT have also been recognized [47].

Bacterial outer membrane vesicles (MVs) are secreted by Gram-negative bacteria with particle sizes ranging from 20 to 400 nm that participate in diverse biological processes, including horizontal gene transfer, the export of cellular metabolites, and cell-to-cell communication [48]. MVs can serve as a delivery system for antibiotic resistance genes. Studies [49] have shown that *Acinetobacter baumannii* can deliver drug resistance genes through MVs, and recent reports also demonstrated that the beta-lactamase gene can transfer to *Escherichia coli (E. coli)* by MVs [50].

Bacterial drug resistance is mainly transmitted by horizontal gene transfer, leading to the spread of bacterial drug resistance [51]. Little is known about the spread of the resistance gene HGT in vivo. Horizontal gene transfer of antibiotic resistance genes has mainly focused on in vitro experiments. In addition, the delivery of antibiotic resistance genes to the recipient bacterium may be much more complicated, and the results of in vitro tests may differ from the real situation in vivo. Research [52] indicates that in vivo models might help to investigate the dissemination of clinically relevant antibiotic resistance genes under more realistic conditions than those currently used within in vitro models. Recently, an increasing number of researchers have paid more attention to the in vivo models to study antibiotic resistance gene transfer.

## 4. In Vivo Horizontal Gene Transfer of Bacterial Resistance

### 4.1. Horizontal Transfer of Antibiotic Resistance Genes in the Human Gut Microbiome

HGT frequently occurs among human intestinal flora, and opportunistic pathogens can obtain ARG through HGT, causing major harm to human health [53]. The “human gut microbiome” describes the microorganisms, their genomes, and the environmental conditions of the human intestinal tract. As an important repository of ARGs [54, 55], the human gut microbiota facilitates the HGT of ARGs. There are many types of ARGs in the human gut, and a large number of bacteria and dense mucus layer in the gut also provide a convenient environment for the spread of ARGs [56]. ARGs from opportunistic pathogens can also be found in the genomes of Gram-positive commensal bacteria, suggesting that the HGT of ARGs is ubiquitous in the gut, especially in Firmicutes [57]. Much of the microbiota's genome plasticity is thought to be attributable to horizontal gene transfer (HGT), and the most effective mechanism of which is conjugation, the exchange of plasmids [58].

In the human gut, antibiotic resistance plasmids and integrative and conjugative elements (ICEs) can also be widely transmitted between commensals and opportunistic pathogens [56]. Under normal conditions, HGT was blocked by the commensal microbiota inhibiting contact-dependent conjugation between Enterobacteriaceae [59]. The mammalian gut is mainly colonized by obligate anaerobic bacteria within the phyla Firmicutes and Bacteroidetes [60]. In the normal gut, Enterobacteriaceae are usually present at very low densities (far less than 108 cfu/g), and the low density of Enterobacteriaceae results in a low frequency of efficient binding plasmid transfer or HGT [61].

Research indicated that inflammation of the host and the production of membrane-destabilizing agents have been proposed to promote HGT in the gut [62]. Inflammatory host responses triggered by the gut immune system (in inflammatory bowel disease patients) or by pathogens can suppress the anaerobic microbiota and boost enterobacterial colonization densities [63, 64]. The increasing prevalence of carbapenemases and extended-spectrum beta-lactamase in the opportunistic pathogenic bacteria *E. coli* and *K. pneumoniae* is readily transmitted in Proteobacteria in the gut [65]. In a streptomycin-treated mouse model of *Salmonella* infection, mouse intestinal inflammation promotes the coproliferation of donor and recipient bacteria in the gut [66, 67]. Stecher et al. [59] have reported the highly efficient HGT of a natural S.Tm plasmid to resident commensal *E. coli* in vivo by using a mouse colitis model and have shown that gut inflammation can boost horizontal gene transfer between pathogenic and commensal Enterobacteriaceae. Research by Crémet et al. [68] revealed that, during a nosocomial outbreak of *Enterobacter cloacae*, there was a possible conjugal transfer of an OXA-48 encoding plasmid from *E. cloacae* to other members of the Enterobacteriaceae in patient's intestines, and then it can spread to other patients. Another study [69] reported the presumable transfer of a multidrug resistance plasmid from *Klebsiella pneumoniae* to *E. coli* in the gastrointestinal tract of a patient. The transfer of resistance genes has been shown in the gastrointestinal tracts, including strains of *Enterococcus faecium* in the gastrointestinal tracts of streptomycin-treated mice and gnotobiotic mice [70].

### 4.2. Horizontal Transfer of Antibiotic Resistance Genes in Animal Models

Many investigations studying HGT are mostly in vitro conditions, but these studies may not represent the real natural environment present in the patients. Several in vivo animal models have been used to study the horizontal dissemination of drug resistance genes in vivo, such as insects, mice, and aquatic organisms. Animal models may mimic more closely the situation in humans than the standard in vitro assays. Insects (*Galleria mellonella*) and mammals (mice) have become the ideal surrogate organism for studying virulence and in vivo evaluation of antibiotic efficacy [71, 72]. The new wax moth larva model is a useful preliminary model for assessing the in vivo efficacy of horizontal gene transfer between species and genera agents before proceeding to mammalian studies, which may reduce the cost of experimentation [73]. And the mammal models can further evaluate the results of the larval models.

Göttig et al. [74] studied the in vivo horizontal gene transfer (HGT) employing the *Galleria mellonella* and low complexity microbiota mice, which found the intergenic gene transfer of OXA-48 in vivo higher transmission frequencies versus in vitro liquid mating experiments. In addition, Price et al. evaluated the effects of clustered regularly interspaced short palindromic repeats (CRISPR-Cas) on the spread of antibiotic resistance in the mouse gastrointestinal model and under different in vitro conditions. And the results showed that CRISPR-Cas antiplasmid activity in vivo was much more obvious than that in vitro experiment conditions [75]. These results demonstrated that in vitro experiments may not appropriately reflect the HGT of the antibiotic resistance gene in vivo.

The research studied that the conjugative transfer of *Salmonella typhi* drug resistance plasmid was also easily transmitted to *Escherichia coli* in mice [76, 77], which provided an essential experimental basis for *Escherichia coli* existing in the animal intestine as a reservoir of drug resistance genes. Lester et al. [78] showed that in the intestine of streptomycin-treated mice, aminoglycoside and macrolide resistance was transferred via conjugation among *Enterococcus* strains.

The spread of drug-resistant genes has been listed as a new type of environmental pollutant [79]. Surface water is a huge reservoir of drug-resistant bacteria and genes [80–82]. The unique living environments enable aquatic animals to easily ingest antibiotic-resistant bacteria (ARB) in water [83]. The gut of aquatic animals is an important place for bacterial growth and reproduction, and at the same time, a large number of native flora colonizing the gut can serve as potential recipient bacteria. Therefore, it may be an important place for the transfer and spread of drug resistance genes in aquatic animals [84]. The gut of fish, as an important aquatic animal, would be a suitable environment for the transfer of antibiotic resistance genes [85]. The spread of ARGs is attributed to horizontal gene transfer such as conjugation, transformation, and transduction. Conjugation is likely to be an important mechanism in the gut because of the surface-contact prevalent in guts [86]. A study [87] explored the transfer rule of bacterial drug resistance genes in zebrafish by constructing a transfer model of drug resistance genes in vivo and demonstrated that drug resistance genes had been transferred and expanded in the zebrafish gut.

### 4.3. Horizontal Transfer of Antibiotic Resistance Genes among Humans, Animals, and the Environment

Bacterial resistance to antimicrobial agents is becoming increasingly common and serious [88]. Animal pathogens are one of the main reservoirs of various drug resistance genes, and they can be continuously transmitted to humans through the food chain, becoming a major hidden danger to public safety [89]. In addition, the natural environment provides a natural drug resistance genes pool for microorganisms, and human activities, environmental changes, and animal migration may all affect the evolution of bacteria and produce new drug resistance genes [90, 91]. Antibiotics have become one of the most frequently detected new pollutants in the environment, and the spread of antibiotics in humans, animals, and the environment has become a research hotspot at home and abroad. Tenhagen et al. [92] and Lozano et al. [93] reported that MRSA can transfer methicillin resistance to humans through milk and food. Plasmid-mediated colistin-resistant strains carrying the mcr-1 gene were first isolated in Chinese animals in 2015, and subsequently, the mcr-1 gene was also detected in humans and the environment, which suggested that Enterobacteriaceae bacteria carrying mcr-1 can adapt well to a variety of hosts and spread between the environment, animals, and humans [94]. Food animals may be a pooled reservoir of resistant bacteria and related resistance genes [95]. With the possibility of antibiotic resistance spreading from livestock and contaminated meat products to people, plant-based foods are fundamental to the food chain of meat-eaters [96]. Recent studies have shown that environmental bacteria colonized in plant-based foods can serve as a platform for the horizontal gene transfer of drug resistance genes. Liao et al. [97] found that fresh lettuce carries beta-lactam-resistant *E. coli* may be a reservoir of resistance genes that could be transmitted to pathogens that cause human infection. A study by Maeusli et al. [96] demonstrated that HGT of antibiotic resistance can occur from *Acinetobacter* to *Escherichia coli (E. coli)* resistance on lettuce. Moreover, transformant *E. coli* from plant experiments can colonize the mouse gut microbiome.

## 5. Factors Influencing the Transfer of Antibiotic Resistance Genes

### 5.1. Antibiotics

As a huge reservoir of antibiotic resistance genes, the human gut microbiome may be involved in the spread of resistance genes to pathogens [98]. External intake of antibiotics or resistance genes may affect the resistance changes of intestinal flora. The irrational use of clinical antibiotics is the main reason for the production of intestinal ARGs, and the long-term clinical use of antibiotics makes the corresponding ARGs in the intestines more abundant [99]. Jakobsson et al. [100] found that the level of the macrolide resistance gene ermB in the gut increased by 3 to 5 orders of magnitude after the subjects received antibiotic treatment. In addition to inducing ARG production, antibiotics can also promote ARG transmission. Wu et al. [101] found that levofloxacin could induce transformation and promote the spread of drug-resistant *E. coli*. Misuse of antibiotics alters gut microbiota homeostasis and promotes horizontal transfer of resistance genes in vivo [98]. The antimicrobial treatment enhances the selection of resistant strains and results in an increase in the resistance gene pool, which ultimately raises the risk of spreading resistance genes [102].

### 5.2. The Restriction-Modification (RM) System and Antirestriction-Modification (Anti-RM) System

**Figure 2:**
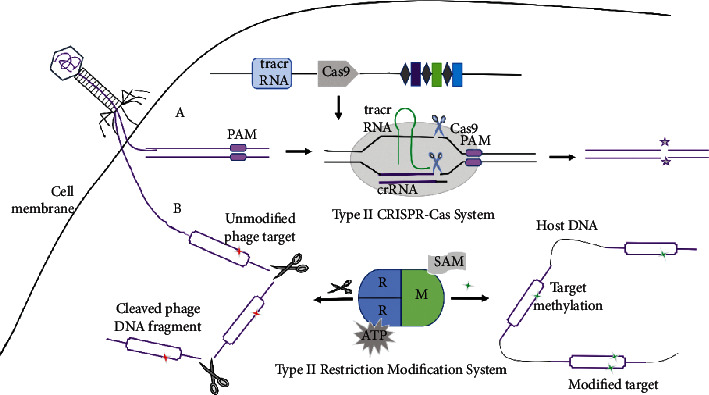
The mechanism of type II CRISPR-Cas system (a) and type II restriction-modification system (b).

Antibiotic resistance gradually increases with the horizontal transfer of mobile elements encoding resistance genes. The RM system plays an important role in regulating the horizontal gene transfer of mobile genetic elements. Restriction-modification (RM) system is a defense system that exists widely in bacteria and archaea [103]. In bacteria, the restriction-modification (RM) system is ubiquitous and is often considered to be the most primitive immune system of bacteria to defend against foreign DNA, such as plasmids or bacteriophages [104]. See Figure 2(a). Restriction endonuclease (REase) specifically recognizes foreign DNA and then cuts and degrades it. Methyltransferase (MTase) methylates modifies its DNA so that it is free of being degraded [105]. RM systems can be divided into four categories: type I, type II, and type III REases with no need for specific sequence methylation for DNA cleavage and type IV REases requiring exogenous methylation models for DNA cleavage [106]. However, the RM system is a major but incomplete barrier to HGT, and antirestriction proteins such as ArdA, ArdB, ArdC, ArdD, and KlcA136 have antirestriction activity and probably facilitate HGT during transduction [107–110]. The RM system in the receptor and the antirestriction system in the mobile genetic element are crucial factors affecting HGT [111].

### 5.3. CRISPR-Cas (Clustered Regularly Interspaced Short Palindromic Repeats (CRISPRs) and CRISPR-Associated Proteins) System and Anti-CRISPR Protein (ACP)

CRISPR-Cas systems serve as an adaptive immune defense system that can defend against invading exogenous genetic material [112]. See Figure 2(b). The CRISPR-Cas system has been found to utilize nucleases programmed with small RNAs to direct sequence-specific cleavage of nucleic acids, prevent the spread of plasmids and phages, and therefore limit horizontal gene transfer mediated by these mobile genetic elements [113–115]. CRISPR-Cas systems confer adaptive immunity against mobile genetic elements that are hypothesized to be a natural impediment to the spread of antibiotic resistance genes [116].

Horizontal gene transfer (HGT) is the main cause of bacterial resistance. The acquired immune defense of the bacterial CRISPR system limits the horizontal transfer of drug resistance genes, thus making bacteria sensitive to antimicrobial drugs to a certain extent [117]. In the study of *Staphylococcus aureus*, it was found that the CRISPR-Cas system can limit the horizontal transfer of bacterial drug resistance genes and prevent the spread of drug resistance genes among staphylococci [118]. A study [119] has shown that multidrug-resistant enterococci lack CRISPR/Cas system elements, suggesting that the CRISPR/Cas system in bacteria may play an important role in hindering drug resistance transmission. The literature suggests an inverse relationship between the occurrence of the type II CRISPR-Cas system and antibiotic resistance in *Enterococcus faecalis* [120].

The CRISPR-Cas system, which provides adaptive immunity to mobile genetic elements (MGEs) in bacteria, is considered a barrier to bacterial horizontal gene transfer and the spread of antibiotic resistance genes [121, 122]. Studies have shown that the CRISPR-Cas system blocks conjugative plasmids to disseminate antibiotic resistance genes among pathogens in vivo. Genetic analysis showed that CRISPR-Cas is a potent barrier to the horizontal acquisition of antibiotic resistance in *E. faecalis*. Price et al. [75] demonstrated that CRISPR-Cas from mammalian intestinal flora can block the in vivo spread of antibiotic resistance plasmids in the mouse intestinal colonization model. Another study [113] showed that the *E. faecalis* CRISPR3-Cas system interferes with the conjugative acquisition of pAM714. A study by Wu and coworkers found that the CRISPR-Cas9 systems target the tetracycline resistance gene (tetM) and erythromycin resistance gene (ermB), respectively, successfully reducing antibiotic resistance to *E. faecalis* in vitro and in vivo [122].

To combat this immune response generated by CRISPR-Cas systems, many phages have evolved anti-CRISPR proteins that inhibit CRISPR-Cas targeting [123]. The anti-CRISPR protein (ACP) complex includes proteins encoded by a variety of mobile genetic elements (MGEs) that inhibit the function of the CRISPR-Cas system at different stages [124, 125] and thereby promoting horizontal gene transfer to a certain degree. However, as pointed out by Stanley et al., the phage encoding anti-CRISPRs remain sensitive to CRISPR-Cas, suggesting that anti-CRISPR action may be an imperfect process.

## 6. Discussion

Antibiotic resistance is spreading rapidly around the world and poses a critical threat to public health [126]. Resistance genes can be transmitted in humans, animals, and the environment, increasing the risk of ingesting resistance genes in humans [127]. There is an urgent need to develop strategies to control multidrug-resistant (MDR) bacterial infections and the spread of antimicrobial resistance. Both horizontal transmissions of bacterial resistance genes and antimicrobial abuse can cause an increase in the proportion of resistant bacteria in the environment [128]. Therefore, it is important to control the use of antimicrobial drugs, thereby alleviating the formation of bacterial drug resistance and slowing down the transmission of bacterial drug resistance.

At present, most antimicrobial drugs already have corresponding drug-resistant bacteria, but as long as new antibiotics are developed faster than the rate of drug resistance generation, then the threat of bacterial infection will be greatly reduced. Therefore, while adopting other measures to deal with bacterial drug resistance, new antimicrobial drugs should also be developed. The study by Ling et al. [129] has developed a new antimicrobial drug called teixobactin, which inhibits bacterial cell wall synthesis by binding to a highly conserved sequence of lipid II and lipid III, and no corresponding resistant bacteria of teixobactin have been found. Furthermore, there are currently reports of graphene oxide (GO) nanocomposites as an antimicrobial agent used to treat infections with multidrug-resistant bacteria [130].

In addition, CRISPR-Cas is an efficient and accurate tool for genome DNA editing [131]. Currently, CRISPR-Cas has been developed as a novel antimicrobial agent to induce bacterial death by specifically targeting and eliminating the antibiotic resistance genes [132, 133]. CRISPR-Cas systems act as adaptive immune systems in bacteria and significantly affect the spread of antibiotic resistance genes and phage infection [134]. Dong et al. [135] constructed the conjugative CRISPR/Cas9 system targeting the mobile colistin resistance gene (mcr-1) in *Escherichia coli*; this engineered CRISPR/Cas9 system can not only eliminate drug-resistant plasmids and resensitize to antibiotics but also make the recipient cell acquire immunity against mcr-1. The CRISPR-Cas system can specifically recognize and target the genetic elements carrying drug resistance genes or their transcripts and limit the spread of drug resistance genes, which shows great potential for preventing and controlling bacterial drug resistance [123]. However, antimicrobial therapy based on CRISPR-Cas technology is still focused on the level of in vitro research, a few in vivo studies have not reached the degree of in vitro research effect, and they are affected by many factors. The clinical treatment of this technique also requires more intensive in vivo research, mainly the application of the complex environment and the host immune response [136].

Even after eliminating the resistance plasmids in the bacteria, the bacteria can continue to uptake the resistance genes from the environment. Therefore, removing a large number of drug resistance genes in the environment and reducing the frequency of drug resistance genes can better delay the current severe situation of bacterial drug resistance.

## 7. Conclusion

The emergence of antibiotic-resistant genes is recognized as a major global health problem. Genetic material-based antibiotic resistance genes (ARGs) mainly can be acquired through gene mutation or horizontal gene transfer and endow the host with antibiotic resistance, thus seriously threatening human health [137, 138]. ARG becomes active due to HGT. HGT of ARGs may lead to the emergence of multidrug-resistant strains. Plasmid conjugation, phage transduction, and natural transformation of extracellular DNA all allow genetic material to jump between strains and species [16, 139, 140]. Conjugative transfer of plasmids is regarded as the most essential way of transferring ARGs between bacteria [141]. The spread of conjugative transfer studies was mostly conducted in vitro, and it was found that the antibiotic resistance genes could be transferred between a variety of bacteria, but it was affected by various factors such as the species and number of bacteria, the size, and temperature of the plasmid. Also, the new antimicrobial agent CRISPR-Cas prevents the spread of antibiotic resistance mostly at the level of in vitro research. Therefore, it is significant to intensively study the spread of antibiotic resistance, investigate the effects of CRISPR-Cas systems, and limit the spread of antibiotic resistance in vivo research. Studying the spread of clinically relevant antibiotic resistance genes under more realistic conditions for in vivo models is crucial for future developing innovative strategies to reduce the spread of bacterial resistance.

In the present study, we briefly elucidated the dissemination mode of drug resistance genes and the mechanism of horizontal gene transmission in bacteria, described the spread of antibiotic resistance genes in in vivo model, outlined the influencing factors that affect the transmission of antibiotic resistance genes, and discussed the countermeasures to bacterial drug resistance, and meanwhile, the future research direction of antimicrobial resistance gene removal is proposed to provide some reference for the control of bacterial drug resistance.

## Data Availability

All data included in this study are available upon request by contacting the corresponding author.
